# Emerging Role of (Endo)Cannabinoids in Migraine

**DOI:** 10.3389/fphar.2018.00420

**Published:** 2018-04-24

**Authors:** Pinja Leimuranta, Leonard Khiroug, Rashid Giniatullin

**Affiliations:** ^1^A.I. Virtanen Institute for Molecular Sciences, University of Eastern Finland, Kuopio, Finland; ^2^Neurotar Ltd., Helsinki, Finland; ^3^Neuroscience Center, University of Helsinki, Helsinki, Finland; ^4^Laboratory of Neurobiology, Kazan Federal University, Kazan, Russia

**Keywords:** migraine, cannabinoids, CGRP, nociception, marijuana, cannabinoid receptor, TRPV1

## Abstract

In this mini-review, we summarize recent discoveries and present new hypotheses on the role of cannabinoids in controlling trigeminal nociceptive system underlying migraine pain. Individual sections of this review cover key aspects of this topic, such as: (i) the current knowledge on the endocannabinoid system (ECS) with emphasis on expression of its components in migraine related structures; (ii) distinguishing peripheral from central site of action of cannabinoids, (iii) proposed mechanisms of migraine pain and control of nociceptive traffic by cannabinoids at the level of meninges and in brainstem, (iv) therapeutic targeting in migraine of monoacylglycerol lipase and fatty acid amide hydrolase, enzymes which control the level of endocannabinoids; (v) dual (possibly opposing) actions of cannabinoids via anti-nociceptive CB1 and CB2 and pro-nociceptive TRPV1 receptors. We explore the cannabinoid-mediated mechanisms in the frame of the Clinical Endocannabinoid Deficiency (CECD) hypothesis, which implies reduced tone of endocannabinoids in migraine patients. We further discuss the control of cortical excitability by cannabinoids via inhibition of cortical spreading depression (CSD) underlying the migraine aura. Finally, we present our view on perspectives of Cannabis-derived (extracted or synthetized marijuana components) or novel endocannabinoid therapeutics in migraine treatment.

## Introduction

Migraine is a debilitating disorder most commonly characterized by a unilateral hemicranial pulsating headache often accompanied by a great variety of other symptoms such as sensory disturbances and nausea (Pavlovic et al., 2014; Russo, 2016). The full list of migraine criteria is provided in the latest version of Headache Classification (ICHD-3 beta, 2013). Due to its high prevalence and disruptive nature, the mechanisms contributing to migraine headache have been intensely studied over many decades but remain debatable. The current consensus states that migraine pain is caused by lowering of the threshold of nociceptive signal processing in response to release of pro-inflammatory agents. Migraine attack’s initiation has been linked to both environmental and hormonal triggers (Pavlovic et al., 2014), which lead to pathophysiological changes due to a sterile neurogenic inflammation in meninges and activation of trigeminal sensory nerves (Pietrobon and Moskowitz, 2013; Gouveia-Figueira et al., 2017).

The multifaceted nature of migraine makes it difficult to define the exact criteria for clinical assessment, and may underlie the vast variability in the ways in which migraine patients respond to existing modes of treatment. Additionally, many of the anti-migraine therapies carry adverse effects, a challenge which has caused discontinuation of research and development of potential anti-migraine drugs (Russo, 2015). For these reasons, introduction of new, more inclusive and effective modes of therapy is urgently needed.

Different parts of *Cannabis sativa* plant have been utilized for centuries in treatment of multitude of health conditions, and consumption of this plant is often associated with psychotropic effects such as mood fluctuations, intoxication, euphoria, increased heart rate, physical dependence upon long-term use, and cognitive impairment (Niyuhire et al., 2007). Regarding migraine pathology, the vital characteristics justifying the proposed use of medical cannabis include anticonvulsive (Rosenberg et al., 2015), analgesic, antiemetic (Parker et al., 2011), and anti-inflammatory effects (Nagarkatti et al., 2009). Mainly due to their potent analgesic action, marijuana-derived exogenous cannabinoids are currently being used for symptomatic and prophylactic treatment in many pain conditions (Oláh et al., 2017), including migraine-associated pain (Chakrabarti et al., 2015). The use of exogenous cannabinoids has been greatly debated as a mode of therapy during past years, but the recent changes in legislation have facilitated their use in several countries. Following the push by the public for increasing cannabinoid availability, the demand for research on cannabinoid substances has also escalated.

This review aims to take a look at the recent publications on the effectiveness and safety of cannabinoid-based migraine treatment, as well as studies of the mechanisms underlying therapeutic effects of these compounds. Based on our experience in experimental studies of migraine, we discuss our own and other available data on the potential applications of cannabinoid therapy in migraine treatment.

## Endocannabinoid System: Exogenous and Endogenous Agonists

Endocannabinoid system (ECS) is a comprehensive signaling system present in virtually every cell type and playing a critical role in maintaining body homoeostasis (Aizpurua-Olaizola et al., 2017). ECS’ numerous components include the enzymes responsible for synthesis of endocannabinoids (eCBs), specific receptors of eCBs, and the post-activity neutralizing pathways (Marco et al., 2012). Here we provide only a short overview of this complex system related to discussion of migraine pathology.

**FIGURE 1:**
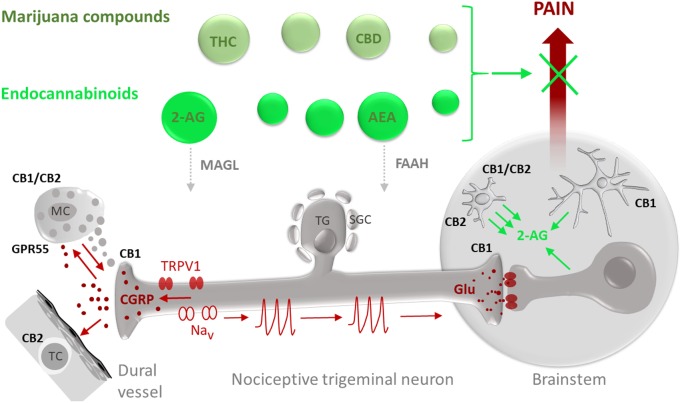
Potential targets for the anti-nociceptive action of phyto-cannabinoids (marijuana compounds) and eCBs in migraine pain. Migraine-associated pain (pain propagating pathways are marked in brown) is generated in the TGVS comprising meningeal mast cells (MC), dural vessels and nociceptive trigeminal nerve fibers. Activation of pro-nociceptive TRPV1 receptors in sensory neurons which cell bodies are located in the trigeminal ganglion (TG) surrounded by satellite glial cells (SGC), results in release of the migraine mediator CGRP which can degranulate MC, provide a strong vasodilatory effect and target T-cells (TC). Degranulation of MC is associated with release of multiple pro-inflammatory compounds (5-HT, histamine, cytokines) supporting local neuroinflammation and sensitization of nociceptive fibers. Sensitized nerve fibers, via activation of certain subtypes of sodium channels (Nav), generate nociceptive firing (nociceptive spikes) propagated to the brainstem and, later, to the higher pain centers where this nociceptive traffic is perceived as migraine pain. eCBs and exocannabinoids (marked in green) including anandamide (AEA) and 2-arachidonoylglycerol (2-AG) as well as their exogenous counterparts THC and CBD, respectively, are promising agents to provide the anti-nociception in migraine. The anti-nociceptive effect of eCBs depends on their local concentrations, determined by the balance between the synthesis and degradation as well as availability and subtypes of their target receptors. The degradation of 2-AG is controlled by MAGL whereas hydrolysis of AEA is determined by the activity of FAAH. As some eCBs, such as AEA, can also activate TRPV1 receptors, migraine-associated pain is affected by a delicate balance between anti-nociceptive effects of CBs on specific cannabinoid receptors and pro-nociceptive effects on TRPV1 receptors. At peripheral site in meninges, CB1 receptors expressed in peripheral trigeminal nerve endings can contribute to anti-nociception by reducing probability of spike generation and reducing release of CGRP. Cannabinoid receptor type 1 (CB1) in the CNS is mainly expressed in neurons with predominant presynaptic location providing the inhibitory action of glutamate release in the brainstem. CB1 subtype is also expressed in astrocytes (Metna-Laurent and Marsicano, 2015). Cannabinoid receptor type 2 (CB2) and GPR55 are primarily expressed in cells of immune origin such as peripheral MC or T-cells as well as in microglial cells in the brain. Recent data also suggest heteromerization of CB1 and CB2 receptors in activated microglia. Notably, microglia are much more efficient than astrocytes and neurons (Walter et al., 2003) in producing the major eCB 2-AG which, in a paracrine way, can control neurons and, in autocrine manner, activate microglia.

To date, several major and many less explored components of the ECS have been identified (Chakrabarti et al., 2015). The most prevalent eCBs are 2-arachidonoylglycerol (2-AG) and arachidonoylethanolamine (anandamide, AEA) (**Figure 1**). Overall, 2-AG is considered the primary signaling molecule and is abundantly expressed throughout the brain (Sugiura et al., 2002). The action of eCBs is mimicked by the main pharmacological components of marijuana, namely phytocannabinoids (pCBs), including the psychotropic Δ9-tetrahydrocannabinol (THC) and the non-psychotropic cannabidiol (CBD) (Oláh et al., 2017; **Figure 1**).

The ECS signals are relayed primarily by two receptors: type 1 cannabinoid receptor (CB1), which is one of the most abundant G-protein coupled receptor in the brain (Smith et al., 2017), and type 2 cannabinoid receptor (CB2), which is functionally related to CB1 but is expressed primarily in peripheral tissues (Chakrabarti et al., 2015). Both CB1 and CB2 are natively activated by eCBs 2-AG and AEA, but they also respond to binding of pCBs. Thus, THC is thought to act primarily via its potent activation of CB1 and CB2. The mechanism of action is less clear for CBD, which has been reported to affect more than 65 discrete molecular targets and to have varied effects outside of ECS (Bih et al., 2015).

One important issue remaining unsolved is how exactly eCBs are released from cells. The traditional dogma states that bioactive eCBs, unlike other neurotransmitters such as acetylcholine and dopamine, are produced “on-demand” (Marsicano et al., 2003). An alternative view suggests that eCBs may be pre-synthesized and stored, much like neurotransmitters (Maccarrone et al., 2010; Fonseca et al., 2013; Chakrabarti et al., 2015).

Endocannabinoid system is active in stress-responsive parts of central and peripheral nervous system, functioning to reduce pain and to alleviate neurodegenerative and inflammatory damage (Preedy, 2017; Smith et al., 2017). Short-term effects induced by eCBs have been shown to involve plastic changes in many brain areas affecting pain sensation (Oláh et al., 2017). All these mechanisms are linked, directly or indirectly, to the migraine pathology.

## Mapping ECS Effects in Migraine Models – Central Vs. Peripheral

The importance of the trigeminovascular system (TGVS) in migraine pathophysiology is widely recognized by experts in the field. During a migraine attack, prolonged activation of the TGVS – comprising meningeal trigeminal nerves and vessels along with dural mast cells (MC) (**Figure 1**) — ultimately causes sensitization of higher order neurons in the central nervous system (CNS), leading to the persistent nociceptive signaling (Burstein et al., 2015). Furthermore, the resulting sensitization has been found to stimulate TGVS activity, creating a positive feedback loop (Eroli et al., 2017). The main migraine mediator associated with the TGVS system is the neuropeptide calcitonin gene-related peptide (CGRP), which promotes vasodilation and contributes to the sterile meningeal inflammation associated with sensitization of nociceptive pathway (Giniatullin et al., 2008; Villalón and Olesen, 2009; Pietrobon and Moskowitz, 2013; Dux et al., 2016; **Figure 1**). All three key meningeal structures (nerves, vessels and MC) can act as targets for the action of pCBs or eCBs. Several papers from P. Goadsby lab have shown that CGRP-induced dilation of dural blood vessels and neuronal pro-nociceptive activity could be reduced by AEA (Akerman et al., 2003, 2007). MC, populating the TGVS in large quantities and responding to CGRP with degranulation (**Figure 1**), likely play a triggering role in migraine (Levy, 2010; Kilinc et al., 2017). In particular serotonin, a major component of mast cell granules, is able to produce a robust activation of trigeminal afferents in meninges (Kilinc et al., 2017; **Figure 1**). Notably, eCB operating via CB1 receptors can stabilize MC (Sugawara et al., 2012) and this effect also contributes to the anti-migraine action of these compounds. However, other data suggest a role for CB2 and the orphan receptor GPR55 in the stabilizing action of cannabinoids on mast cell HMC-1 line (Cantarella et al., 2011). It is yet to be studied using migraine models, but similar mast cell stabilizing effect in meninges could potentially contribute to the anti-migraine action of cannabinoids (**Figure 1**).

Cannabinoid effects on the CNS are mediated primarily by inhibitory CB1 receptors, located throughout CNS as well as in afferent neurons (Marco et al., 2012). Both within CNS and peripherally, eCBs act as retrograde messengers or synaptic modulators for their respective target cells (Gabral et al., 2015). Thus, one of the main functions of the eCB 2-AG, degraded by the enzyme monoacylglycerol lipase (MAGL, Aaltonen et al., 2016), is to serve as a mediator of retrograde signaling to downregulate neurotransmitter release (Smith et al., 2017). Unlike the selective presynaptic inhibitory effect of adenosine on excitatory glutamatergic terminals (Safiulina et al., 2005), activation of CB1 receptor by eCB inhibits the release from presynaptic terminals of both inhibitory and excitatory neurotransmitters (Gabrielli et al., 2015; Iseger and Bossong, 2015).

CB2 receptor, being, like CB1 receptor, highly sensitive to 2-AG, possesses an individual set of expression patterns and characteristic functions. Thus, CB2 expression is higher in peripheral organs than in the CNS and is mostly restricted to the immune system cells including B and T lymphocytes (**Figure 1**). Endocannabinoid system contributes to both innate and adaptive immune responses, functioning as a preventative force against the onset of pro-inflammatory responses (Nagarkatti et al., 2009; Oláh et al., 2017). CB2 receptors are primarily responsible for exerting immunosuppressive effects in the periphery. During an inflammatory reaction, which is expected in most severe or chronic forms of migraine, more of CB2 receptors are made available for activation (Gabral et al., 2015). In our recent study, familial migraine was found to be associated with enhanced concentrations of key inflammatory cytokines detected in blood (Khaiboullina et al., 2017). Thus, cannabinoids may act by correcting the dysregulation of cytokine production (Nagarkatti et al., 2009). Taken together, these studies suggest that the less explored CB2 receptors possessing the anti-inflammatory potential (Gabral et al., 2015) represent a promising target to counteract migraine (Scherma et al., 2016).

Besides their independent functions, CB1 and CB2 receptors have been shown to work together by forming hetero-receptor complexes (Callen et al., 2012). This type of receptor-receptor interaction has been shown recently for brain-residing immune cells such as microglia. Thus, it has been recently shown that, alongside CB2 receptors, the CB1-CB2 heteroreceptor complexes are expressed in microglia (Smith et al., 2017; Navarro et al., 2018; **Figure 1**). Microglia could play a part in the pathogenesis of migraine with aura, since the cortical spreading depression (CSD) associated with this type of migraine effectively activates microglia (Shibata and Suzuki, 2017). CSD also releases ATP (Karatas et al., 2013), which is a major driver of microglia, promoting release of the eCB 2-AG (Walter et al., 2003). Notably, the ability of microglia to secrete 2-AG is about 20-times higher than that of astrocytes and neurons (Walter et al., 2003). In view of the recent data, this link appears to be even more intriguing as microglia are essential for initiation of CSD (Pusic et al., 2014). Interestingly, this positive feedback loop could be disrupted by agonists of CB1 (but not of CB2 receptors), which block CSD (Kazemi et al., 2012). Consistent with growing interest to the medications targeting receptor heteromers, a study using the bivalent CB1 antagonist specifically affecting dimerized CB1 receptors, showed pain-alleviating effects (Zhang et al., 2010). Overall, di- and oligomerization of GPCRs within CNS represent an attractive therapeutic target in pain conditions (Borroto-Escuela et al., 2013; Fuxe and Borroto-Escuela, 2015).

In peripheral migraine mechanisms, activation of TRPV1 receptor, a non-selective cation channel expressed in trigeminal nociceptors, leads to massive CGRP release (Kageneck et al., 2014; **Figure 1**). Our and other studies indicate an important contribution of TRPV1 receptors to migraine pathology (Zakharov et al., 2015; Dux et al., 2016). Stimulation of dural sensory nerves by capsaicin was found to cause vasodilation modulated by CGRP via TRPV1 receptor (Dux et al., 2016). As the TRPV1 channels can also bind eCB AEA (Chakrabarti et al., 2015), this may result in unwanted pro-nociceptive action of cannabinoids, causing neuroinflammation in meninges. This complexity may explain why increased doses of cannabinoids diminished their analgesic effect (Kandasamy et al., 2018). It further creates an incentive for development of new synthetic CBs with minimal activity on TRPV1 receptors, or specific MAGL inhibitors, which, apart from triggering the accumulation of anti-nociceptive 2-AG, can decrease the level of the pro-nociceptive arachidonic acid (AA) and reduce pain (Aaltonen et al., 2016). MAGL inhibitors may also reduce the pro-nociceptive downstream products of AA such as endovanilloids, agonists of TRPV1 receptors (Hwang et al., 2000). Interestingly, the inhibition of fatty acid amide hydrolase (FAAH) degrading AEA is also anti-nociceptive in migraine models (Greco et al., 2015).

## Clinical Endocannabinoid Deficiency (CECD) Hypothesis

Endocannabinoid system’s role in homeostatic upkeep highlights the importance of this system in maintaining overall health. Disruptions in supply or functionality of eCB ligands have been connected to numerous mental state disturbances and, particularly, to migraine. Migraine, along with comorbid conditions such as fibromyalgia and irritable bowel syndrome, share symptomatic commonalities of hyperalgesia as well as treatment resistance, likely stemming from common pathophysiological phenomenon: CECD. The CECD hypothesis suggests a correlation between deficient levels of eCB and pain (Russo, 2016).

Since the initial proposal of the CEDC in 2001, the importance of maintaining regular eCB levels was shown in a study comparing CB1- and CB2-KO mice that experienced inflammation, to mice lacking FAAH (and thus having elevated AEA) with reduced inflammation responses (Oláh et al., 2017). The lowered inhibitory activity of eCS in migraine, possibly due to reduced CB1 and CB2 receptor expression, serves as an assertion for the compensatory therapy with exogenous cannabinoids. According to the CECD hypothesis, treatment of migraine using exogenous cannabinoids could be achieved with low doses due to predisposition for elevated neuronal excitability. The CECD-causing deficiencies can appear for congenital reasons, or can be acquired.

## PRO and Contra of Cannabinoids in Migraine Treatment

There is a long history of using cannabinoids for effective treatment of pain conditions. Due to their long-standing status of out-lawed substances (Baron, 2015), it is worth taking a look at the arguments still standing in the way of legalization. Overall, targeting ECS with peripherally acting drugs is a promising strategy for development of safe migraine treatments. However, there are still many insufficiently explored issues that may be detrimental for this seemingly harmless treatment.

Regularly experiencing chronic migraine pain can have adverse impacts on social relationships and job status which can lead into psychological distress (Ramsden et al., 2015). As it stands, the first ‘pro’ is that the treatment with pCB can acutely alleviate the resulting stress, in addition to tackling the initial cause by pain reduction.

In a study of the cannabis use for self-medication in Germany, Austria and Switzerland, 10.2% of patients reported using it for migraine headache symptoms (Kandasamy et al., 2018). Another group found that the self-treatment outcome was highly variable, with low doses tending to alleviate migraine while higher doses even triggering headaches (Lu and Anderson, 2017). These findings call for creating a highly specific prescription for individual patients, which would be required for safe and successful treatment plan.

One of the main problems arising from the long-term usage of cannabis is the physical reliance on the pCBs, mainly THC. Moreover, there is evidence that patients can develop tolerance for pCBs (Kandasamy et al., 2018). Carelessly establishing a reliance on any form of medication may carry more ill effects on the patient’s mentality, and may even lead to weakening or loss of pain relief.

A crucial point when considering cannabinoid treatment is that smoking marijuana is the most common method of pCB self-administration. When self-administering pCBs via smoking, the relief seekers often use marijuana mixed with tobacco leaves. In view of the recently established crosstalk between nicotinic cholinergic and ECS (Scherma et al., 2016), the nicotinic cholinergic system has been proposed as a molecular target for treating cannabis dependence (Scherma et al., 2016). Particularly interesting is the ability of the endogenous nicotinic antagonist kynurenic acid to counteract the addictive effects of CBs (Justinova et al., 2013). Notably, new derivatives of kynurenic acid were suggested recently as promising medicines for migraine (Greco et al., 2017) opening a new perspective for combined CB+antinicotinic therapy of this disorder. Interestingly, the main migraine mediator CGRP can reduce the activity of nicotinic receptors (Giniatullin et al., 1999), suggesting that the migraines associated with enhanced level of endogenous CGRP are ‘pre-conditioned’ to respond better to CB treatments.

Cannabidiol (CBD), the second most prevalent pCB, should also be explored in relation to migraine treatment. Unlike THC with its characteristic CB1 receptor affinity, CBD does not have intoxicating and psychoactive effects linked with CB1 receptor activation. Yet, CBD possesses anxiolytic (anxiety-reducing) and antipsychotic properties that have been suggested to be inflicted via interactions with TRPV1 and non-endocannabinoid GPR55 receptor (Bih et al., 2015). Recently, the US Food and Drug Administration (FDA) accepted an application for Epidiolex^®^ (active agent CBD) in treatment of seizures prominent in Lennox-Gastaut syndrome (LGS) and Dravet syndrome (GW Pharmaceuticals, 2017). This stands as an important milestone paving the way for possible repurposing of this CBD-based drug for treating migraine, as well as other related neurological conditions.

## Conclusion

In summary, cannabinoids – due to their anticonvulsive, analgesic, antiemetic, and anti-inflammatory effects – present a promising class of compounds for both acute and prophylactic treatment of migraine pain. In view of the rapidly unfolding changes in the legal status of cannabis, research on (endo)cannabinoids has become pertinent once again. Formal approval of a cannabinoid-based drug for other pathologies opens a possibility for repurposing these agents also to treat migraine. The abundance of CB1 receptors in the brain makes them an attractive target for treatment of migraine via blocking not only peripheral but also the central nociceptive traffic and reducing the pathologically enhanced cortical excitability predisposing to CSD. CB2 receptors in immune cells can be targeted to reduce the inflammatory component associated with severe forms of migraine. Exogenous compounds lacking the unwanted peripheral pro-nociceptive component or eCBs generated via inhibited degradation pathways and combined with other supportive agents are most desirable for this aim. Moreover, primary stratification of patients to identify and predict the effectiveness of cannabinoid treatment can greatly improve the efficiency of this approach.

## Author Contributions

PL, LK, and RG wrote the article and designed the figure.

## Conflict of Interest Statement

LK is a founder and stake-holder of the company Neurotar LTD. The other authors declare that the research was conducted in the absence of any commercial or financial relationships that could be construed as a potential conflict of interest.
